# Digital coping of parents in conflict-affected communities: a path to maintain health and well-being: Stav Shapira

**DOI:** 10.1093/eurpub/ckac131.167

**Published:** 2022-10-25

**Authors:** S Shapira, D Yeshua Katz, O Braun-Lewensohn

**Affiliations:** 1 School of Public Health, Ben-Gurion University of the Negev, Beer Sheva, Israel; 2 Department of Social Work, Ben-Gurion University of the Negev, Beer Sheva, Israel; 3 Conflict Resolution and Conflict Management Program, Ben-Gurion University of the Negev, Beer Sheva, Israel

## Abstract

**Background:**

Mobile apps such as WhatsApp have become significant resources for information and social support in times of crisis. Little is known about the role WhatsApp groups play in the context of living in conflict-affected regions. Living in such areas is associated with myriad mental health impacts, and recent studies have identified parents of young children as highly vulnerable in this regard. The study’s aim was to examine parents’ digital coping with political violence in southern Israel.

**Methods:**

In-depth interviews were conducted with 21 parents of young children (<17) residing in communities near the Israel-Gaza border and who are members of local online parents’ groups on WhatsApp. Data were analyzed to answer questions regarding the benefits and disadvantages that parents assign to online groups; and regarding the role of digital communication in maintaining health and well-being.

**Results:**

The findings reveal that online groups are often discussed as a shared and ubiquitous coping resource that supports mental health and well-being. Parents report they use the group to share ways to deal with the situation effectively and exchange social support, and as a space that facilitates community cohesion. Furthermore, during escalations, members perceived the group as the most trustworthy source of information. The groups’ continuous availability had drawbacks as well. Some participants pointed to digital stress caused by the fear of missing out on information, or by information overload.

**Conclusions:**

Our results demonstrate the effectiveness of digital environments in helping parents cope with a collective stressor: a particularly worthy goal given that social media apps now constitute the primary online connection for most individuals. This has broad impacts on health promotion efforts for mental health issues related to political violence as well as other large-scale health crises such as the COVID-19 pandemic.

**Key messages:**

• Digital environments can serve as a platform for successful coping with collective stressors.

• Health practitioners should consider drawbacks when planning digital support efforts.

